# Supplementary material for: Factors explaining resilience among nepalese nurses of tertiary-level hospital experiencing COVID-19 pandemic: A cross-sectional study
Source: PLOS Ment Health. 2025 Nov 12;2(11):e0000468. doi: 10.1371/journal.pmen.0000468 (PMC12798480; doi:10.1371/journal.pmen.0000468)
Supplement: S1 Table — (DOCX) [file pmen.0000468.s001.docx]

**S1 Table. Mean, standard deviation, skewness, and kurtosis of each item of the resilience**

| **S. N.** | **Statements** | **Before Multivariate Outlier Management**  **(*N* = 307)** | | | | **After Multivariate Outlier Management of Aggregate Scores (*N* = 288)** | | | |
| --- | --- | --- | --- | --- | --- | --- | --- | --- | --- |
|  |  | ***M*** | ***SD*** | **Skewness** | **Kurtosis** | ***M*** | ***SD*** | **Skewness** | **Kurtosis** |
|  | Ability to adapt | 3.19 | .79 | -.78 | .59 | 3.20 | .79 | -.80 | .64 |
|  | Can deal with whatever comes | 3.19 | .82 | -.98 | .91 | 3.25 | .74 | -.90 | 1.11 |
|  | Try to see the humorous side of things | 2.33 | 1.11 | -.27 | -.53 | 2.36 | 1.09 | -.31 | -.42 |
|  | Cope with stress | 2.87 | .91 | -.74 | .37 | 2.89 | .88 | -.71 | .41 |
|  | Tend to bounce back | 2.85 | .91 | -.63 | .06 | 2.85 | .91 | -.63 | .09 |
|  | Believe the ability to achieve | 3.29 | .75 | -1.05 | 1.18 | 3.35 | .65 | -.73 | .52 |
|  | Stay focused and think clearly | 3.12 | .77 | -.77 | .82 | 3.17 | .67 | -.36 | -.28 |
|  | Am not easily discouraged | 2.98 | .85 | -.57 | -.07 | .2.99 | .84 | -.58 | .01 |
|  | Think of myself as a strong person | 3.25 | .73 | -.88 | .85 | 3.27 | .70 | -.81 | .73 |
|  | Able to handle | 3.03 | .84 | -.73 | .46 | 3.07 | .78 | -.58 | .24 |
