# Supplementary material for: Factors explaining resilience among nepalese nurses of tertiary-level hospital experiencing COVID-19 pandemic: A cross-sectional study
Source: PLOS Ment Health. 2025 Nov 12;2(11):e0000468. doi: 10.1371/journal.pmen.0000468 (PMC12798480; doi:10.1371/journal.pmen.0000468)
Supplement: S2 Table — (DOCX) [file pmen.0000468.s002.docx]

**S2 Table. Mean, standard deviation, skewness, and kurtosis of each item of the self-efficacy**

| **S. N.** | **Statements** | **Before Multivariate Outlier Management**  **(*N* = 307)** | | | | **After Multivariate Outlier Management of Aggregate Scores (*N* = 288)** | | | |
| --- | --- | --- | --- | --- | --- | --- | --- | --- | --- |
|  |  | ***M*** | ***SD*** | **Skewness** | **Kurtosis** | ***M*** | ***SD*** | **Skewness** | **Kurtosis** |
|  | I can always manage to solve difficult problems if I try hard enough. | 3.61 | .52 | -.80 | -.63 | 3.61 | .52 | -.82 | -.55 |
|  | If someone opposes me, I can find the means and ways to get what I want. | 3.17 | .69 | -.71 | .94 | 3.16 | .68 | -.68 | 1.09 |
|  | It is easy for me to stick to my aims and accomplish my goals. | 3.08 | .73 | -.34 | -.43 | 3.11 | .71 | -.28 | -.55 |
|  | I am confident that I could deal efficiently with unexpected events. | 3.25 | .63 | -.41 | .13 | 3.27 | .62 | -.43 | .27 |
|  | Thanks to my resourcefulness, I know how to handle unforeseen situations. | 3.24 | .61 | -.19 | -.56 | 3.26 | .60 | -.17 | -.53 |
|  | I can solve most problems if I invest the necessary effort. | 3.62 | .54 | -1.01 | -.04 | 3.64 | .52 | -1.00 | -.13 |
|  | I can remain calm when facing difficulties because I can rely on my coping abilities. | 3.31 | .64 | -.38 | -.69 | 3.33 | .63 | -.39 | -.67 |
|  | When I am confronted with a problem, I can usually find several solutions. | 3.22 | .63 | -.28 | -.21 | 3.23 | .61 | -.27 | -.09 |
|  | If I am in trouble, I can usually think of a solution. | 3.41 | .60 | -.57 | -.08 | 3.43 | .59 | -.59 | -.01 |
|  | I can usually handle whatever comes my way. | 3.34 | .65 | -.54 | -.32 | 3.34 | .64 | -.53 | -.27 |
