# Supplementary material for: Factors explaining resilience among nepalese nurses of tertiary-level hospital experiencing COVID-19 pandemic: A cross-sectional study
Source: PLOS Ment Health. 2025 Nov 12;2(11):e0000468. doi: 10.1371/journal.pmen.0000468 (PMC12798480; doi:10.1371/journal.pmen.0000468)
Supplement: S3 Table — (DOCX) [file pmen.0000468.s003.docx]

**S3 Table. Mean, standard deviation, skewness, and kurtosis of each item of the perceived social support**

| **S. N.** | **Statements** | **Before Multivariate Outlier Management**  **(*N* = 307)** | | | | **After Multivariate Outlier Management of Aggregate Scores (*N* = 288)** | | | |
| --- | --- | --- | --- | --- | --- | --- | --- | --- | --- |
|  |  | ***M*** | ***SD*** | **Skewness** | **Kurtosis** | ***M*** | ***SD*** | **Skewness** | **Kurtosis** |
|  | There is a special person who is around when I am in need. | 5.01 | 1.43 | -.94 | .41 | 5.06 | 1.39 | -.94 | .48 |
|  | There is a special person with whom I can share my joys and sorrows. | 5.39 | 1.40 | -1.30 | 1.13 | 5.47 | 1.33 | -1.33 | 1.35 |
|  | My family really tries to help me. | 5.78 | 1.21 | -1.77 | 3.54 | 5.88 | 1.06 | 1.06 | 3.31 |
|  | I get the emotional help and support I need from my family. | 5.83 | 1.25 | -1.72 | 3.08 | 5.91 | 1.14 | 1.14 | 3.22 |
|  | I have a special person who is a real source of comfort to me. | 5.65 | 1.37 | -1.55 | 2.32 | 5.75 | 1.22 | 1.22 | 2.48 |
|  | My friends really try to help me. | 5.26 | 1.14 | -1.02 | 1.58 | 5.31 | 1.10 | 1.10 | 1.90 |
|  | I can count on my friends when things go wrong. | 4.87 | 1.23 | -.81 | .55 | 4.94 | 1.19 | 1.19 | .71 |
|  | I can talk about my problems with my family. | 5.67 | 1.21 | -1.55 | 2.81 | 5.77 | 1.08 | 1.08 | 2.78 |
|  | I have friends with whom I can share my joys and sorrows. | 5.24 | 1.25 | -1.09 | 1.27 | 5.33 | 1.14 | 1.14 | 1.63 |
|  | There is a special person in my life who cares about my feelings. | 5.63 | 1.38 | -1.48 | 1.92 | 5.76 | 1.22 | 1.22 | 2.36 |
|  | My family is willing to help me make decisions. | 5.71 | 1.24 | -1.55 | 2.69 | 5.80 | 1.12 | 1.12 | 3.09 |
|  | I can talk about my problems with my friends. | 5.26 | 1.26 | -1.12 | 1.58 | 5.36 | 1.15 | 1.15 | 1.87 |
