# Supplementary material for: Factors explaining resilience among nepalese nurses of tertiary-level hospital experiencing COVID-19 pandemic: A cross-sectional study
Source: PLOS Ment Health. 2025 Nov 12;2(11):e0000468. doi: 10.1371/journal.pmen.0000468 (PMC12798480; doi:10.1371/journal.pmen.0000468)
Supplement: S4 Table — (DOCX) [file pmen.0000468.s004.docx]

**S4 Table. Mean, standard deviation, skewness, and kurtosis of each item of the perceived organizational support**

| **S. N.** | **Statements** | **Before Multivariate Outlier Management**  **(*N* = 307)** | | | | **After Multivariate Outlier Management of Aggregate Scores (*N* = 288)** | | | |
| --- | --- | --- | --- | --- | --- | --- | --- | --- | --- |
|  |  | ***M*** | ***SD*** | **Skewness** | **Kurtosis** | ***M*** | ***SD*** | **Skewness** | **Kurtosis** |
|  | This hospital administration values my contribution to its well-being. | 3.88 | 1.80 | -.89 | -.16 | 3.99 | 1.70 | -.95 | .14 |
|  | The hospital administration fails to appreciate any extra effort from me. ^a^ | 3.20 | 1.95 | -.01 | -1.24 | 3.21 | 1.92 | -.01 | -1.20 |
|  | The hospital administration would ignore any complaint from me. ^a^ | 3.32 | 1.93 | -.19 | -1.22 | 3.34 | 1.90 | -.19 | -1.20 |
|  | The hospital administration really cares about my well-being. | 3.23 | 1.93 | -.30 | -1.04 | 3.30 | 1.90 | -.33 | -.97 |
|  | Even if I did the best job possible, the hospital administration would fail to notice. ^a^ | 3.50 | 1.99 | -.25 | -1.23 | 3.50 | 1.99 | -.23 | -1.23 |
|  | The hospital administration cares about my general satisfaction at work. | 3.31 | 1.85 | -.33 | -.92 | 3.33 | 1.82 | -.33 | -.90 |
|  | The hospital administration shows very little concern for me. ^a^ | 3.26 | 1.90 | -.14 | -1.08 | 3.27 | 1.89 | -.11 | -1.07 |
|  | The hospital administration takes pride in my accomplishments at work. | 3.74 | 1.80 | -.59 | -.53 | 3.79 | 1.77 | -.62 | -.43 |

***Note.*** ^a^: Reversed item.
