# Supplementary material for: Factors explaining resilience among nepalese nurses of tertiary-level hospital experiencing COVID-19 pandemic: A cross-sectional study
Source: PLOS Ment Health. 2025 Nov 12;2(11):e0000468. doi: 10.1371/journal.pmen.0000468 (PMC12798480; doi:10.1371/journal.pmen.0000468)
Supplement: S5 Table — (DOCX) [file pmen.0000468.s005.docx]

**S5 Table. Mean, standard deviation, skewness, and kurtosis of each item of compassion satisfaction**

| **S. N.** | **Statements** | **Before Multivariate Outlier Management (*N* = 307)** | | | | **After Multivariate Outlier Management of Aggregate Scores (*N* = 288)** | | | |
| --- | --- | --- | --- | --- | --- | --- | --- | --- | --- |
|  |  | ***M*** | ***SD*** | **Skewness** | **Kurtosis** | ***M*** | ***SD*** | **Skewness** | **Kurtosis** |
|  | I get satisfaction from being able to help people. | 4.60 | .67 | -2.12 | 6.13 | 4.62 | .64 | -2.17 | 7.15 |
|  | I feel invigorated after working with those I help. | 4.44 | .77 | -1.80 | 4.50 | 4.48 | .73 | -1.83 | 4.98 |
|  | I like my work as a helper. | 4.25 | .90 | -1.43 | 2.38 | 4.30 | .85 | -1.43 | 2.57 |
|  | I am pleased with how I am able to keep up with helping techniques and protocols. | 4.49 | .67 | -1.41 | 2.90 | 4.51 | .85 | -1.43 | 3.15 |
|  | My work makes me feel satisfied. | 4.43 | .77 | -1.30 | 1.42 | 4.45 | .73 | -1.22 | 1.21 |
|  | I have happy thoughts and feelings about those I help and how I could help them. | 4.35 | .72 | -1.06 | 1.50 | 4.35 | .70 | -.98 | 1.35 |
|  | I believe I can make a difference through my work. | 4.37 | .69 | -.82 | .20 | 4.38 | .67 | -.75 | .09 |
|  | I am proud of what I can do to help. | 4.43 | .74 | -1.26 | 1.54 | 4.45 | .72 | -1.32 | 1.90 |
|  | I have thoughts that I am a "success" as a helper. | 4.26 | .86 | -1.42 | 2.48 | 4.27 | .85 | -1.46 | 2.82 |
|  | I am happy that I chose to do this work. | 4.54 | .68 | -1.49 | 2.44 | 4.56 | .66 | -1.52 | 2.63 |
