# Supplementary material for: Factors explaining resilience among nepalese nurses of tertiary-level hospital experiencing COVID-19 pandemic: A cross-sectional study
Source: PLOS Ment Health. 2025 Nov 12;2(11):e0000468. doi: 10.1371/journal.pmen.0000468 (PMC12798480; doi:10.1371/journal.pmen.0000468)
Supplement: S6 Table — (DOCX) [file pmen.0000468.s006.docx]

**S6 Table. Mean, standard deviation, skewness, and kurtosis of each item of burnout**

| **S. N.** | **Statements** | **Before Multivariate Outlier Management (*N* = 307)** | | | | **After Multivariate Outlier Management of Aggregate Scores (*N* = 288)** | | | |
| --- | --- | --- | --- | --- | --- | --- | --- | --- | --- |
|  |  | ***M*** | ***SD*** | **Skewness** | **Kurtosis** | ***M*** | ***SD*** | **Skewness** | **Kurtosis** |
|  | I am happy. ^a^ | 1.72 | .75 | 1.01 | 1.55 | 1.72 | .71 | .82 | .87 |
|  | I feel connected to others. ^a^ | 2.02 | .92 | .65 | .10 | 2.00 | .90 | .70 | .31 |
|  | I am not as productive at work because I am losing sleep over the traumatic experiences of a person I help. | 2.49 | 1.12 | .39 | -.42 | 2.47 | 1.12 | .45 | -.34 |
|  | I feel trapped by my job as a helper. | 2.11 | 1.16 | .82 | -.17 | 2.08 | 1.13 | .82 | -.12 |
|  | I have beliefs that sustain me. ^a^ | 1.68 | .90 | 1.42 | 1.94 | 1.64 | .87 | 1.47 | 2.16 |
|  | I am the person I always wanted to be. ^a^ | 1.77 | .87 | 1.10 | 1.07 | 1.74 | .84 | 1.04 | .88 |
|  | I feel worn out because of my work as a helper. | 2.90 | 1.27 | .13 | -.91 | 2.84 | 1.24 | .16 | -.83 |
|  | I feel overwhelmed because my case [work] load seems endless. | 3.01 | 1.06 | -.01 | -.14 | 3.00 | 1.05 | .01 | -.11 |
|  | I feel "bogged down" by the system. | 2.15 | 1.24 | .93 | -.13 | 2.10 | 1.20 | .97 | .03 |
|  | I am a very caring person. ^a^ | 1.61 | .68 | .99 | 1.40 | 1.59 | .68 | 1.06 | 1.57 |

***Note.*** ^a^: Reversed item.
