# Supplementary material for: Factors explaining resilience among nepalese nurses of tertiary-level hospital experiencing COVID-19 pandemic: A cross-sectional study
Source: PLOS Ment Health. 2025 Nov 12;2(11):e0000468. doi: 10.1371/journal.pmen.0000468 (PMC12798480; doi:10.1371/journal.pmen.0000468)
Supplement: S7 Table — (DOCX) [file pmen.0000468.s007.docx]

**S7 Table. Mean, standard deviation, skewness, and kurtosis of each item of secondary traumatic stress**

| **S. N.** | **Statements** | **Before Multivariate Outlier Management**  **(*N* = 307)** | | | | **After Multivariate Outlier Management of Aggregate Scores**  **(*N* = 288)** | | | |
| --- | --- | --- | --- | --- | --- | --- | --- | --- | --- |
|  |  | ***M*** | ***SD*** | **Skewness** | **Kurtosis** | ***M*** | ***SD*** | **Skewness** | **Kurtosis** |
|  | I am preoccupied with more than one person I help. | 3.90 | .87 | -.25 | -.71 | 3.90 | .86 | -.21 | -.70 |
|  | I jump or am startled by unexpected sounds. | 3.53 | 1.04 | -.07 | -.83 | 3.53 | 1.04 | -.06 | -.88 |
|  | I find it difficult to separate my personal life from my life as a helper. | 2.80 | 1.12 | .20 | -.53 | 2.79 | 1.11 | .27 | -.47 |
|  | I think that I might have been affected by the traumatic stress of those I help. | 2.54 | 1.13 | .36 | -.52 | 2.53 | 1.14 | .40 | -.49 |
|  | Because of my help, I have felt "on edge" about various things. | 2.16 | 1.13 | .69 | -.24 | 2.16 | 1.13 | .73 | -.13 |
|  | I feel depressed because of the traumatic experiences of the people I help. | 2.75 | 1.06 | .17 | -.32 | 2.73 | 1.05 | .16 | -.33 |
|  | I feel as though I am experiencing the trauma of someone I have helped. | 3.69 | .97 | -.32 | -.41 | 3.72 | .97 | -.31 | -.46 |
|  | I avoid certain activities or situations because they remind me of the frightening experiences of the people I help. | 2.55 | 1.22 | .42 | -.73 | 2.53 | 1.21 | .46 | -.64 |
|  | As a result of my helping, I have intrusive, frightening thoughts. | 2.37 | 1.17 | .62 | -.38 | 2.37 | 1.16 | .64 | -.31 |
|  | I can't recall important parts of my work with trauma victims. | 2.70 | 1.19 | .35 | -.61 | 2.69 | 1.17 | .37 | -.58 |
