# Supplementary material for: Factors explaining resilience among nepalese nurses of tertiary-level hospital experiencing COVID-19 pandemic: A cross-sectional study
Source: PLOS Ment Health. 2025 Nov 12;2(11):e0000468. doi: 10.1371/journal.pmen.0000468 (PMC12798480; doi:10.1371/journal.pmen.0000468)
Supplement: Attachment — Submitted filename: Letter Addressing Reviewers Comments 8-2-2025.docx [file pmen.0000468.s009.docx]

**Date:** 08-02-2025

To,

The Chief Editor,

PLOS Mental Health

**Subject:** Cover Letter

**Dear Madam/Sir,**

On behalf of the Principal Investigator and team, I extend a sincere gratitude to the editors and all the anonymous reviewers for giving us this opportunity to revise and refine our manuscript. We did our best based on the comments. We have submitted the required supplementary materials, including the raw data with all personal identifiers excluded, within the supplementary file. Now, I wish to submit our revised research article entitled “Factors Explaining Resilience Among Nepalese Nurses of Tertiary-level Hospital Experiencing COVID-19 Pandemic: A Cross-sectional Study” for consideration by PLOS Mental Health. We confirm that this work is original and has not been published, nor is it currently under consideration for publication elsewhere. Furthermore, we request you to provide us an opportunity with constructive comments for improving and clarifying the concepts of our interest. We would like to assure you that we will do our best to meet the standards of this journal.

We have no conflicts of interest to disclose. I would like to request that you address all correspondence concerning this manuscript to me at rekhatimalsina@pahs.edu.np. Thank you so much for your consideration of our manuscript.

**Yours Sincerely,**

Dr. Rekha Timalsina,

Associate Professor,

Patan Academy of Health Sciences,

School of Nursing and Midwifery,

Sanepa, Lalitpur, Nepal

| **Editor’s Comments** | **Revised and Highlighted with Yellow Color** |
| --- | --- |
| We note that you have indicated that data from this study are available upon request. PLOS only allows data to be available upon request if there are legal or ethical restrictions on sharing data publicly. | We have submitted the required supplementary materials, including the raw data with all personal identifiers excluded, within the supplementary file. |
